# Effects of spike-time dependent plasticity on deep brain stimulation of the basal ganglia for treatment of Parkinson's disease

**DOI:** 10.1186/1471-2202-16-S1-P83

**Published:** 2015-12-18

**Authors:** Logan L Grado, Matthew D Johnson, Theoden I Netoff

**Affiliations:** 1 Graduate Program in Biomedical Engineering, University of Minnesota, Minneapolis, MN 55455, USA; 2 Institute for Translational Neuroscience, University of Minnesota, Minneapolis, MN 55455, USA

## 

Deep brain stimulation (DBS) of the basal ganglia is a widely used and effective treatment for patients with medication-refractory Parkinson's disease (PD). However, tuning the stimulation parameters to maximize therapy while minimizing side effects is performed mostly through a trial and error approach, consuming time and energy of both clinician and patient [1]. As such, there is a need for a systematic, engineering based approach to improve patient outcomes. Current theories of DBS mechanisms propose that DBS suppresses pathological oscillations (15-35 Hz) that dominate the basal ganglia by suppressing information flow [2,3]. However, effects of DBS are not instant, often taking minutes or more before an effect is seen [4]. This time scale suggests DBS may induce a change in network architecture through synaptic plasticity, destabilizing oscillations in the network. Recently, a new approach to DBS called "Coordinated Reset" appears to take advantage of this phenomenon, resulting in therapeutic benefits that last from hours to days [5,6]. We hypothesize that if beta oscillations are indeed responsible for Parkinsonian signs, then the dissipation and return of these oscillations should follow a time course similar to that of the symptoms themselves. We use a computational network model of PD with emergent pathological 34 Hz oscillation developed by Hahn & McIntyre [7] to test the effects of DBS on the basal ganglia, implemented with spike-time dependent plasticity (STDP) as described by Badoual et al. [8]. Preliminary results show that with the introduction of STDP, pathological beta oscillations dissipate over time after the onset of DBS stimulation in computational models. This work suggests that it may be possible to tune stimulation settings to take advantage of long term plasticity effects in DBS to improve patient outcomes.
